# Network-Exposure Severity and Self-Protective Behaviors: The Case of COVID-19

**DOI:** 10.1093/geroni/igab015

**Published:** 2021-05-11

**Authors:** Howard Litwin, Michal Levinsky

**Affiliations:** Israel Gerontological Data Center, Paul Baerwald School of Social Work and Social Welfare, The Hebrew University of Jerusalem, Mount Scopus, Israel

**Keywords:** Pandemic, Personality traits, SHARE, Social network

## Abstract

**Background and Objectives:**

To clarify whether awareness of the extent and severity of exposure to the coronavirus disease 2019 (COVID-19) in the social networks of older adults is related to the engagement by the latter in self-protective behaviors. The inquiry is guided by the Health Belief Model and by concepts from the domain of social networks.

**Research Design and Methods:**

Data from the Survey of Health, Ageing and Retirement in Europe (SHARE) were used, including the SHARE COVID-19 Survey executed in the summer of 2020. The study sample numbered 33,053 persons aged 50 and older in 26 countries. We regressed a logged count of self-protective behaviors on network-exposure severity, controlling for sociodemographic background, country, personality traits, and self-exposure severity. Age and network-exposure interaction terms were examined, as were “close family” and “other” network ties.

**Results:**

Network-exposure severity was positively associated with the extent of engagement in self-protective behaviors among older adults, but mainly among the oldest group, aged 70 and older. Awareness of exposure severity in “close family” and “other” networks were similarly associated with self-protection. Respondents from countries with the lowest rates of COVID-19 infection at the time (Latvia, Finland, and Denmark) engaged in fewer self-protective behaviors, while those from countries with high infection rates (Spain, Italy, and Portugal) self-protected to a greater degree.

**Discussion and Implications:**

The study findings point to the role of the social network, even if indirect, in promoting self-protective behaviors among the oldest segment of society. Policymakers should collaborate with the social networks of older adults in order to promote the adoption of self-protective behaviors. Such intervention might help to reduce the threat of infection among the most vulnerable age group.

**Translational Significance:** We found that the more that older people know about the extent of COVID-19-related symptoms, testing, illness, and death within their social networks, the more they engage in self-protective behaviors. Because older adults constitute the group that is the most vulnerable to the ill effects of COVID-19, it is important to encourage their adoption of self-protective behaviors as a means to reduce the risk of infection. Policymakers and practitioners should work proactively through the social networks of older adults toward this end. Positive and supportive communications through the social network may be more effective than general announcements in the media.

This study looks at the social correlates of engagement in self-protective behaviors among older adults in the period that followed the initial outbreak of the coronavirus disease 2019 (COVID-19) pandemic. Self-protective behavior is an essential individual means for resisting infections (Lee et al., 2019). Immediately after the outbreak of COVID-19, therefore, public health experts promoted the adoption of enhanced adherence to self-protective measures (Lep et al., 2020). It was hoped that increased personal hygiene and social distancing would “flatten the curve” (Zickfeld et al., 2020), that is, reduce the spread of COVID-19.

Research indicates, however, that self-protective behaviors are not equally adopted among different groups or in different settings (Liu et al., 2019). The Health Belief Model (HBM) maintains that individual health-related actions are differentially shaped by demographic variables and psychological characteristics (particularly, personality), and that these factors work through a number of pathways, among them perceived susceptibility, severity, benefits, and barriers (Glanz & Bishop, 2010). Analyses have shown, for example, that the HBM is appropriate in describing self-protective behavior in the context of pandemic influenza (Durham et al., 2012).

Another conceptual framework that informs the current study is the domain of social networks. The construct of social network reflects the interpersonal environment in which people are embedded and from which they may derive a range of benefits (Litwin, 1996). An informative paradigm put forward by Berkman and colleagues (2000) shows that social networks work through four key pathways: (a) provision of social support, (b) social influence, (c) social engagement, and (d) access to resources and material goods. The authors note, moreover, that the “social influence which extends from the network’s values and norms constitutes an important and under-appreciated pathway through which networks impact health” (p. 849).

In the present study, we examine whether awareness of exposure to aspects of the COVID experience among those in one’s social network is a social influence that affects one’s health-related practices. We define the notion of network exposure as knowing people who have had any level of COVID-like symptoms. The term does not refer, in our study, to infection-inducing physical contact with members of the network. That is, we are interested in the social influence that the presence of COVID in one’s social network has on one’s engagement in self-protective behaviors.

Our analytic model focuses on the pathway of perceived severity in the HBM in relation to the social network. Specifically, we consider network-exposure severity, which we define as the extent of the social network that has experienced the COVID-19 virus in any of its various aspects (e.g., symptoms, testing, and hospitalization). To the best of our knowledge, this variable has not been studied in relation to self-protective behavior. The first hypothesis to be tested in the analysis, therefore, is that greater exposure to COVID-19 among the members of one’s social network is positively associated with the extent of one’s own engagement in self-protective behaviors.

Networks vary in terms of relationship type and roles, as for example kin ties and friendship ties (Fiori et al., 2008). Social networks also vary in terms of the strength of the respective ties, but it is unclear whether strong social ties are more influential for health than weak social ties are (Kauppi et al., 2018). Moreover, both family ties and other types of ties, for example, friends or neighbors, may be strong or weak (Mair & Thivierge-Rikard, 2010). Given these distinctions, we pose a tentative second study hypothesis: greater exposure to COVID-19 among close family members (presumed to be strong ties) is more positively associated with the extent of one’s own engagement in self-protective behaviors than is exposure to COVID-19 among friends, neighbors, and others (presumed to be weak ties).

As noted above, the HBM holds that demographic variables affect health behaviors through the designated pathways. The main such variable of interest to the present study is age. Several recent studies addressed the association of age and self-protective behaviors in the COVID era. A survey in Australia of people aged 18 and older found that there was less health-protective behavior among the younger ages (Faasse & Newby, 2020), as was found in a Turkish sample as well (Yıldırım et al., 2021). In a study of persons aged 18 and older in Germany, the associations with age were inconsistent or unrelated (Lüdecke & von dem Knesebeck, 2020). A study in Portugal revealed that engagement in protective behaviors declines with advancing age (Pasion et al., 2020), and an analysis of an American convenience sample showed that older men implemented the fewest behavior changes compared to the others (Barber & Kim, 2021). Given the lack of conclusive findings in this area, we examine a general research question in the current inquiry: Is age related to engagement in self-protective behaviors among older adults?

As for other relevant demographic factors, a meta-analysis of the association between gender and respiratory epidemic-related protective behaviors found that women were more likely to adopt what the authors call “nonpharmaceutical behaviors” (Moran & Del Valle, 2016). Higher education was related to greater self-protective behavior in Norway (Zickfeld et al., 2020) and, correspondingly, lower education correlated with less self-protective behavior in Germany (Lüdecke & von dem Knesebeck, 2020). Data from the ELSI-COVID-19 initiative in Brazil indicated that multimorbidity was associated with protective behavior (Batista et al., 2020). Finally, a paper on the severe acute respiratory syndrome outbreak in Taiwan reported that self-protective behavior was significantly associated with marital status (Chuo, 2014). Based upon this brief review, the current analysis controls for all of these factors.

The second main predictive block of variables in the HBM is that of psychological characteristics. We focus, in this respect, on the role of personality. Much of the research in this area in the past decades has used the Big Five framework of personality traits (McCrae & John, 1992). The model distinguishes between five dominant traits or attributes: (a) openness to experience, (b) conscientiousness, (c) extraversion, (d) agreeableness, and (e) neuroticism (Roccas et al., 2002).

Research suggests that there are personality characteristics that predispose the development of patterns of health behavior (Bermudez, 1999). Conscientiousness reflects the propensity to be self-controlled and goal-directed. It is understandable, therefore, why this trait may correlate with the self-protective outcome. Neurotic individuals see things as being more severe, and hence their possibly greater engagement in COVID-related self-protective behavior. Openness is related to intelligence and insight, which may promote engagement in self-protection (Erlich & Litwin, 2019).

In the context of the COVID-19 pandemic, one American study found, for example, that neuroticism and openness were positively associated with COVID-19 anxiety (Nikčević et al., 2021). In terms of self-protective behavior, a study in Qatar reported that conscientiousness and neuroticism predicted social distancing (Abdelrahman, 2020). The results of a recent online survey also indicated that social distancing was predicted by personality traits, in this case: agreeableness and conscientiousness (Blagov, 2021). Given these sundry findings, the current analysis controls for the five personality attributes.

As noted earlier, the HBM posits perceived severity as a key pathway through which background demographic variables and personality traits affect health behaviors. The current study focuses upon network-exposure severity as the key variable of interest. However, personally perceived and/or experienced severity must be taken into account as well. A study in Taiwan found, for example, that perceived severity was an important factor in motivating individuals’ intentions to adopt influenza protective behaviors such as wearing a mask and washing hands (Gong et al., 2020). In contrast, an online survey of a representative sample of American and Canadian adults discovered an association between the belief that COVID-19 is an exaggerated threat and disregard for social distancing, poor hand hygiene, and antivaccination attitudes (Taylor et al., 2020). Hence, we control for self-exposure, as a measure of personally perceived severity, in our study.

Finally, country effects must also be taken into account. One recent study examining cross-country differences in behavioral responses to the COVID-19 pandemic found that countries with higher levels of reciprocity and trust were related to greater health-protective behavioral responses (Buyukkececi, 2020). However, another study reports that it is still unclear as to how governmental and individual factors interact in relation to protective behaviors against COVID-19 (Dai et al., 2020). Given the state of the literature, we hypothesize that there will be country differences in relation to the extent of engagement in self-protective behaviors. However, due to lack of further evidence, we do not predict, a priori, which differences are expected.

## Research Design and Methods

### Sample

This investigation uses data from the Survey of Health, Ageing and Retirement in Europe (SHARE), a panel study of adults aged 50 and older (Börsch-Supan et al., 2013), including a telephone survey executed in the summer of 2020 (Scherpenzeel et al., 2020). We also draw upon data from Wave 7 (2018) and Wave 8 (2020). Those with complete data numbered 44,874 persons, from 26 countries (see Figure 2 for the list). The analytical sample excluded spouses under age 50, as well as those missing data on study variables (3,593) or reported they had never left their homes since the outbreak (8,228). The resultant analytical sample was 33,053 participants.

### Variables

First, we constructed a count of *self-protective behaviors* comprised of eight items. Respondents were asked whether they: (a) washed their hands more frequently than usual, (b) used special hand sanitizer or disinfection fluids more frequently than usual, (c) paid special attention to covering cough and sneeze, and/or (d) took drugs or medicine as a prevention against the virus. Responses for each were coded as yes = 1 and no = 0. Two additional probes asked the frequency of: (e) wearing a facemask when going outside the home to a public space, and (f) keeping distance from others when outside the home. We coded the responses such that “often” or “always” = 1, and “sometimes” or “never” = 0. Another two items asked the frequency of: (g) meeting with more than five people from outside the household, and (h) visiting other family members. Responses for these were reverse-coded such that “less often” or “not any more” = 1, and “about the same” or “more often” = 0. The scores on these eight probes were summed creating a count of 0–8 (mean = 6.06, *SD* = 1.22, skewness = −1.32). Because the distribution was skewed, we performed a log transformation of the scores for use in the multivariate stage of the statistical analysis.

The main independent variable was the extent of exposure to COVID-19 in the respondent’s social network. Exposure severity was queried in relation to nine relationship categories: (1) respondent; (2) spouse or partner; (3) parent; (4) child; (5) other household member; (6) other relative outside household; (7) neighbor, friend, or colleague; (8) caregiver; and (9) other. We used a count approach for delineation of the network property of interest, as is used in such surveys as Health and Retirement Study and English Longitudinal Study of Ageing, and in the Lubben Social Network Scale (Lubben et al., 2006). However, we counted only those who were exposed to COVID symptoms, as our aim was to capture the extent of exposure to the COVID-19 experience within the respondent’s interpersonal environment.

For each category, respondents were asked how many people: (a) experienced COVID symptoms (cough, fever, or difficulty breathing); (b) were tested and the result was negative; (c) were tested and the result was positive; (d) were hospitalized due to infection; or (e) died due to the virus (not asked of respondents). We coded the exposures according to severity. One point each was scored for experiencing symptoms and having negative test results, 2 points for positive test results, 5 points for hospitalization, and 10 points for death. Scores on each exposure were multiplied by the number of persons cited in each relationship category and then summed. A large majority of the sample reported having had no exposure whatsoever (72.2%). Moreover, the frequency distribution had a long skew. Consequently, we capped the score scale for each category at 3 points and higher.

A *network*-*exposure severity* variable was created by summing the scores for the relationship categories 2–9 described above. In addition, we calculated two specific network-exposure variables in order to take into account key relationship differences. The variable *close-family*-*exposure severity* summed the scores for categories 2–5, while the variable *other-network*-*exposure severity* did the same for categories 6–9. Finally, a corresponding, *self-exposure severity* variable was created using the same scoring scheme for the respondent’s self-reported experiences (category 1).

Personality characteristics were obtained through the Big Five Personality Inventory (BFI). In Wave 7, SHARE introduced a 10-item version of the inventory (BFI-10) (Rammstedt & John, 2007). The BFI-10 was found to have sufficient validity and reliability in the SHARE data (Levinsky et al., 2019). The respective five personality characteristics used were: *openness*, *conscientiousness*, *extraversion*, *agreeableness*, and *neuroticism.*

The analysis also considered several background characteristics. Stable characteristics (age, gender, education) were retrieved from Wave 7. Varying characteristics (economic status, marital status) were taken from the SHARE COVID-19 Survey, or from Wave 8 if data were missing. Health status was addressed by a self-rated health (SRH) from Wave 7, as well as a postoutbreak update as to whether one’s health has improved, worsened, or stayed about the same.

Age was measured according to three groupings: *mid-life age (50–59)*, *young-old age (60–69)*, and *older age (70+)*. Dichotomous dummy variables were created for each age group. *Gender* (male = 0; female = 1) and *marital status* (no live-in partner = 0, live-in partner = 1) were also addressed as dummy variables. Economic status was measured on a probe of one’s capacity to manage financially (range = 1–4); a higher score reflects better *financial capacity* (Litwin & Sapir, 2009). We considered *education* by means of the Internal Standard Classification of Education 1997 (ISCED-97), in which a higher score reflects higher education (0–6). SRH was examined on a 5-point scale, the higher the score the better one’s health. Worse post-outbreak health was coded as: worsened = 1, stayed about the same or improved = 0. Lastly, *country* of residence was considered by means of dummy variables. An additional country-related variable assigned the cumulative COVID-19 death rates in the respective countries on August 1, 2020, the mid-point of the 3-month data collection period.

### Analysis

The empirical investigation began with univariate description and bivariate examination of the study variables. In the multivariate stage of the analysis, we performed ordinary least square regressions on the log of the dependent variable—self-protective behaviors. Two models were considered. In the first model, the self-protective behaviors score was regressed on the background variables, the personality traits, self-exposure severity, and network-exposure severity. In the second model, we added interaction terms for age group and network-exposure severity. The interaction terms were mean-centered. Supplemental regressions were performed as well, as will be reported. All analyses were executed using STATA 15.

## Results

Table 1 presents a univariate description of the study variables. Self-exposure to COVID was extremely rare among the participants in the sample, and exposure to the virus among the members of their social networks was very limited. The average ranking of engagement in self-protective behaviors, on the other hand, was relatively high.

**Table 1.** Univariate Description of Study Variables (*N* = 33,005) Among Europeans Aged 50 and Older

Variables	Mean	*SD*	Range
Background			
Age			
50–59	0.20	0.40	0/1
60–69	0.36	0.48	0/1
70+	0.44	0.50	0/1
Gender (female)	0.57	0.49	0/1
Education	3.13	1.40	0–6
Financial capacity	2.81	0.99	1–4
Self-rated health—baseline	2.88	1.01	1–5
Worse postoutbreak health	0.08	0.27	0/1
Live-in partner	0.72	0.45	0/1
Big Five personality traits			
Openness	3.35	0.93	1–5
Conscientiousness	4.13	0.79	1–5
Extraversion	3.51	0.92	1–5
Agreeableness	3.67	0.83	1–5
Neuroticism	2.64	1.01	1–5
After COVID-19 outbreak			
Self-exposure severity	0.08	0.34	0–3
Network-exposure severity	0.64	1.33	0–14
Self-protection behaviors	6.06	1.22	0–8

*Notes*: COVID-19 = coronavirus disease 2019.

Table 2 shows that the extent of self-exposure severity and network-exposure severity was positively related to younger age (50–59), higher education, better financial capacity, and worse postoutbreak health, and negatively related to advanced age (70+). Exposure to COVID among the members of the social network correlated with female gender and marital status as well, and it correlated negatively with baseline health. The two exposure types, self and network, were interrelated. Women engaged more frequently than men in self-protective behaviors, as did those with a partner, and those aged 60–69. Adults with better financial status engaged less frequently in self-protective behaviors, as did adults aged 70 and older. The personality traits showed mixed associations with self-protective behaviors, at the bivariate level. The two exposure types, self and network, were positively associated with self-protective behaviors, albeit weakly.

**Table 2.** Bivariate Associations Between Background, Personality Traits, and After-COVID-Outbreak Variables: Pearson Correlations

Variables	After COVID-19 outbreak		
	Self-exposure severity	Network-exposure severity	Self-protective behaviors
	*R*	*R*	*R*
Background			
Age			
50–59	.060***	.062***	.008
60–69	−.004	.009	.035***
70+	−.049***	−.066***	−.043***
Gender (female)	.004	.015**	.085***
Education	.053***	.093***	−.017**
Financial capacity	.051***	.172***	−.063***
Self-rated health—baseline	.002	−.079***	.031***
Worse postoutbreak health	.107***	.022***	.044***
Live-in partner	.009	.047***	.080***
Big Five personality traits			
Openness	.019***	.049***	.021***
Conscientiousness	.001	.025***	.050***
Extraversion	.013**	.041***	−.024***
Agreeableness	.004	.045***	−.025***
Neuroticism	−.006	−.035***	.089***
COVID-19 exposure			
Self-exposure severity		.205***	.025***
Network-exposure severity			.026***

*Notes*: COVID-19 = coronavirus disease 2019.

**p < .01. ***p < .001.

The results of the multivariate analysis are presented in Table 3. Model 1 shows that the extent of self-exposure severity and network-exposure severity remained positively related to one’s engagement in self-protective behaviors, after taking into account the other study variables. As for age, both the younger group (50–59) and the older group (70+) engaged in fewer self-protective behaviors than those aged 60–69.

**Table 3.** Predictors of Self-Protective Behaviors Among Europeans Aged 50 and Older: OLS Regressions

Variables	Self-protective behaviors (log)	
	Model 1 β	Model 2 β
Background		
Age		
50–59^a^	−.022***	−.022***
70+ ^a^	−.013**	−.012**
Gender (female)	.098***	.098***
Education	.060***	.060***
Financial capacity	−.032**	−.032***
Self-rated health—baseline	−.018**	−.018**
Worse postoutbreak health	.029***	.029***
Marital status (live-in partner)	.089***	.089***
Big Five personality traits		
Openness	.013*	.013*
Conscientiousness	.038***	.038***
Extraversion	.009	.009
Agreeableness	.007	.007
Neuroticism	.035***	.035***
COVID-19 exposure		
Self-exposure severity	.020***	.020***
Network-exposure severity	.024***	.011
Interaction terms		
Age 50–59 × Network-exposure severity		.009
Age 70+ × Network-exposure severity		.015*
Observations	33,053	33,053
*R* ^2^	.106	.106

*Notes*: COVID-19 = coronavirus disease 2019. All models control for country; OLS = ordinary least squares.

^a^Reference category: age: 60–69.

*p < .05. **p < .01. ***p < .001.

The regression shows that neurotic and conscientious individuals engaged to a similar degree in self-protective behaviors. Those with greater openness were also associated with self-protective behavior, but to a minor degree. As for the background variables, women, the partnered, and those with higher education engaged more often in self-protective behaviors, as did those with worse post-outbreak health. Better financial capacity, in contrast, was negatively associated.

**Figure 1.**
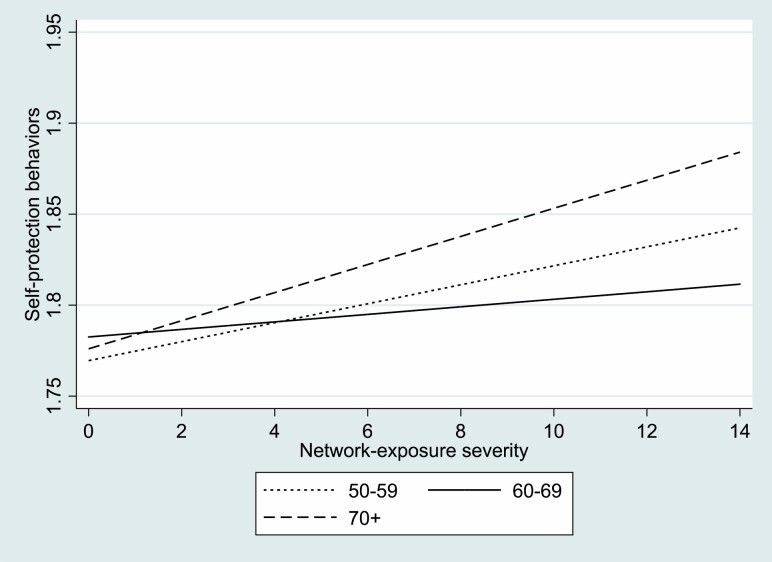
Self-protective behaviors by network-exposure severity according to age grouping.

Model 2 shows that when interacting age group and network-exposure severity, the main effect of the exposure network becomes insignificant, while the interaction with the oldest age group (70+) emerges as significant, albeit weakly. Figure 1 shows the respective slopes. As illustrated, the association between exposure-network severity and self-protective behavior among those aged 60–69 (the reference category in the regression) is almost even. A slight rise is discerned among those aged 50–59, but the steepest relative rise was among adults aged 70 and older. Thus, the association of exposure to COVID-19 among the members of the social network and one’s own extent of engagement in self-protective behaviors was significant only among the oldest group.

**Figure 2.**
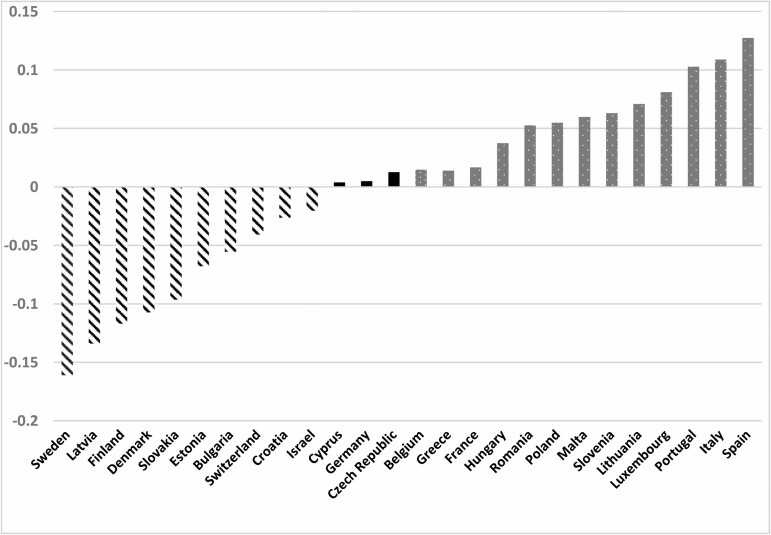
Country deviation from the all-country mean of self-protective behaviors, after controlling for the study variables.

Figure 2 shows the net associations between country and self-protective behaviors. The graph presents the deviation of each country from the all-country mean. The countries on the left side of the graph (indicated by bars with diagonal black and white stripes) engaged in significantly fewer self-protective behaviors and those on the right side (gray dotted bars) had more such behaviors. Those in the middle (solid black bars) did not differ from the overall country mean. Sweden, Latvia, Finland, and Denmark were the countries reporting the least engagement in self-protective behaviors, while Spain, Italy, and Portugal reported the greatest such engagement.

We ran four supplemental analyses (available in Supplementary Material). In the first, we looked at previous social network data on the current study sample, retrieved from SHARE Wave 6 (the last wave for which generated network data are available). We found that close family ties had greater frequency of contact (mean = 2.61) compared to frequency of contact with other network ties (mean = 1.47, *T* = 127.6, *p* < .001), and greater emotional closeness as well (means = 1.72 and 1.21, respectively, *T* = 125.1, *p* < .001). We tentatively conclude, therefore, that close family ties in our sample generally reflect strong ties while other network ties reflect weak ties, for the most part. (We note that we did not include the category of “other relatives in the household” in this examination, as it was not queried in the SHARE Wave 6 data, but this relationship category applied to less than one percent of the current study sample.) 

The next three supplemental analyses were all regressions. In the first, we replaced the network-exposure variable with the two relationship-based network-exposure measures: close-family-exposure severity and other-network-exposure severity. The results of the regression were generally the same as in the previous analysis. As for network exposure, both of the relationship-based network-exposure measures in Model 1 were positively associated with the self-protection behavior outcome (β = .014 and .018, respectively, *p* < .001). In Model 2, only the interaction of older age (70+) and other-network-exposure severity was significant (β = .015, *p* < .05).

In the second supplemental regression, we replaced the country dummies with the cumulative COVID-19 death rates in the respective countries. Here, the other-network-exposure severity variable correlated with self-protective behavior (β = .012, *p* < .05; Model 1), but close-family-exposure severity did not. The interaction of older age (70+) and other-network-exposure severity was close to significance (β = .014, *p* = .05; Model 2), and another interaction term achieved significance (Age 50–59 × Close-network-exposure severity; β = .018, *p* < .05). In this model, however, the explained variance lessened substantially. Similar trends appeared in the third supplemental regression, in which we eliminated country dummies and death rates from the analysis. We note that the inverted-U pattern of age group in relation to self-protective behaviors was upheld in all the models.

## Discussion and Implications

This study sought to clarify whether exposure to the COVID-19 virus within the social networks of older adults was related to self-protective behaviors on the part of the latter during the initial phase of the pandemic. Toward this end, we drew upon aspects of the HBM (Glanz & Bishop, 2010) and concepts from the domain of social networks (Berkman et al., 2000). Our analysis also sought to elucidate whether the association, if confirmed, occurs to the same degree among older people of different ages.

Specifically, we investigated whether network-exposure severity was associated with the extent of engagement in self-protective behaviors, controlling for the effects of sociodemographic background and personality characteristics. The inquiry introduced age/network-exposure interaction terms into the multivariate analysis as well. The aim of the study was not to confirm or refute the HBM, per se. Rather, we utilized selected constructs from the model to inform and to shape the analysis.

The study is based upon a first release of data from the SHARE COVID-19 Survey, launched in the summer of 2020 to capture the initial effects of the pandemic. We also utilized data from previous waves of SHARE (2018, 2020), in order to incorporate the control variables that were central to the model. Some 33,000 respondents from 26 countries comprised the combined study sample.

We note, first, that exposure to the virus was quite limited among the participants in the study, and minimally experienced among those in their social networks as well. This finding suggests that despite the dominant presence of the pandemic in the media and in governmental epidemic-control decisions, the vast majority of older Europeans did not personally experience the virus at the outset of the pandemic. In contrast, the extent of engagement in self-protective behaviors was high, on average. Thus, most people took extra steps to insure their personal safety. Nevertheless, there were also those who adopted few self-protection measures, if at all.

The first hypothesis in the present inquiry was that greater exposure to COVID-19 among the members of one’s social network is positively associated with the extent of one’s own engagement in self-protective behaviors. This hypothesis was confirmed. That is, after taking sociodemographic background and health into account, as well as personality traits and extent of self-exposure to the virus, the network-exposure severity variable retained a small but significant positive correlation.

Social network theory provides a potential explanation for this effect (Litwin, 1996). Individual behaviors are influenced by the feedback received from one’s interpersonal social ties. The members of one’s social network serve as sources of cognitive guidance and social support. As such, the influence of the network on an individual is generally greater than are inputs from unknown or untrusted others. This difference explains why knowing about exposure to the virus among one’s social network may motivate a self-protective response more frequently than does general information about the pandemic in the public domain, disheartening as that information may be.

The HBM adds another caveat through its construct of perceived severity. When the perceived severity of the pandemic is observed among those closest to you, that is, the members of your social network, you are more motivated to react in some way. Hence, greater network-exposure severity is related to greater engagement in self-protective behavior.

Our second hypothesis proposed that greater exposure to COVID-19 among close family members is more positively associated with the extent of one’s own engagement in self-protective behaviors than is exposure to COVID-19 among friends, neighbors, and others. The hypothesis was not confirmed in the current analysis in that both relationship types were found to equally associate with the self-protection outcome. That is, the effect of close-family-exposure severity was not greater than that of the effect of other-network-exposure severity. Thus, such relations as friends and neighbors seem to have as much social influence as close family ties in relation to the association between network-exposure-severity and self-protective behavior.

Next, we asked whether age is related to engagement in self-protective behaviors among older adults. All the regression models showed that both the youngest group (age 50–59) and the oldest group (age 70+) engaged in fewer self-protective behaviors than those aged 60–69. Some recent studies have suggested that older adults engage less in such behavior (Faasse & Newby, 2020; Pasion et al., 2020), but they did not consider age differences within the older cohort. This particular age comparison, therefore, is an innovation of the current analysis.

Our study does not supply a direct explanation for the inverted-U pattern of age group in relation to self-protective behaviors that emerged. We might speculate, however, that young-old persons (those aged 60–69) are more aware of recent or pending age-related changes (Sabatini et al., 2019) and, consequently, engage in greater self-protective behavior. The middle-aged persons (aged 50–59) tend to engage in less self-protective behavior, perhaps due to employment-related exigencies (e.g., the need to meet colleagues, to speak without a face mask, etc.). Older adults (70 and older) also engage in fewer self-protective behaviors. Such behavior may be due to their having lesser access to web-based communications concerning the need for self-protective measures, or perhaps to their having more fatalistic attitudes concerning quality of life and end-of-life decisions.

It might be the case that the inverted-U pattern of age group that emerged in the present study actually reflects cohort rather than life course differences. To examine this possibility, we ran the regression without the country variable and, as noted, found that the inverted-U pattern remained. We tentatively maintain, therefore, that the life course explanation is a plausible one.

The interaction of age and network-exposure severity revealed that their mutual association with self-protective behaviors was significant only among the oldest group. Thus, self-protective individual behavior is seen to be related to what occurs in one’s social network primarily among the oldest adults, those who were likely to have retired years earlier. This finding has significant implications for policy and practice. It underscores that those most at risk of ill-health due to COVID infection, that is, the oldest age group, may underestimate the importance of engaging in self-protective behavior.

However, it also reveals that those of them who encounter the virus within their social networks tend to self-protect to a greater degree. This suggests that any attempt to influence behavior change among older people (i.e., the adoption of self-protective measures) would benefit from intensive outreach directed to their personal social ties. It is not enough to rely upon the news or public broadcasts to increase self-protective behavior among older adults. Personal social networks are an important means through which to promote health-related behavior change.

Although we did not formulate hypotheses in relation to the other control variables, we note that most of the sociodemographic variables acted in accordance with previous findings. This result was true, as well, for the personality characteristics, which showed that conscientiousness and neuroticism were the traits most related to COVID-related self-protective behavior (Abdelrahman, 2020; Blagov, 2021). The trait of openness to experience showed a weak positive association with self-protective behaviors in the current study as well.

Our last study hypothesis predicted country differences in relation to the outcome, net of the effects of the other variables. We found that respondents from several countries (e.g., Sweden, Latvia, Finland, and Denmark) did indeed engage in fewer self-protective behaviors, while those from some other countries (e.g., Spain, Italy, and Portugal) self-protected to a greater degree.

A potential explanation for these differences ties in directly to the HBM. Latvia, Finland, and Denmark (but not Sweden) were among the countries with the lowest cumulative rates of COVID-19-related infection per 100,000 population at the time of the SHARE COVID-19 Survey (summer 2020), while Spain, Italy, and Portugal were among those with the highest cumulative rates per 100,000 population. Thus, perceived severity might be reflected in the relative rates of morbidity due to the pandemic in these particular settings. Those with higher infection rates had greater perceived severity and, consequently, greater self-protective behaviors. Those with lower infection rates tended to engage less in self-protection.

To examine this line of explanation further, we ran a regression in which we replaced the country dummies with the respective cumulative country rates of death from COVID-19. The results revealed that while the death rates were a significant predictor (the higher the death rates, the greater the engagement in self-protective behaviors), the explained variance of the model dropped substantially. This outcome suggests that there are other country differences as well that are expressed in the country dummy variables. We offer one such explanation in the next paragraph.

Among the countries with the lowest extent of self-protection were the Nordic welfare-state nations (Greve, 2007). People in those societies may have maintained a high degree of trust in the societal mechanisms that managed the pandemic. Therefore, they relaxed their own degree of self-protection. The countries with the greatest extent of self-protection, on the other hand, were Spain, Italy, and Portugal. They are classified as Southern welfare states (Rhodes, 1996), in which families are the prime supports and there are less established collective social mechanisms. A greater need for self-reliance, in those societies, might explain their greater engagement in COVID-related self-protective behaviors. Further inquiry into the country differences is clearly warranted.

We should acknowledge a few limitations of the present study. First, the data for this analysis were drawn from a first release. Further treatment of the data might modify the associations that were found here. However, given the urgency of the COVID-19 crisis and the need to understand its implications, as soon as possible, this may be a minor shortcoming. A second limitation is that the extent of self and network exposure to COVID-19 was somewhat sparse in the sample at the time of the survey. The low rate of exposure may partly account for the weak (albeit significant) correlations that emerged. It is quite likely, however, that the rates of exposure have increased since then. The SHARE project will administer a follow-up telephone survey in mid-2021, allowing further warranted research in this area. A third limitation is that we did not take into account specific epidemic-control measures that may have been implemented in different countries. This limitation is minimized, nevertheless, by the fact that country differences were controlled in the multivariate analysis.

In conclusion, this study points to the role of the social network, even if indirect, in promoting self-protective behaviors among the oldest cohort. Such behaviors take on critical importance in times of crisis, and especially in the era of COVID-19. Networks seem to promote the adherence to self-protective measures of older adults when the network members themselves are ill or at risk. Because social networks are an important conduit through which health practices are shaped and adopted, policymakers and practitioners should find a way to work proactively through the social ties of older adults. That is, ways need to be found to encourage the adoption of self-protective behaviors through positive and supportive communications from the social network. Such intervention might help to reduce the threat of infection among those in the most vulnerable age group.

## Supplementary Material

igab015_suppl_Supplementary_Material
