# Supplementary material for: Network-Exposure Severity and Self-Protective Behaviors: The Case of COVID-19
Source: Innov Aging. 2021 May 11;5(2):igab015. doi: 10.1093/geroni/igab015 (PMC8136077; doi:10.1093/geroni/igab015)
Supplement: igab015_suppl_Supplementary_Material [file igab015_suppl_supplementary_material.docx]

Supplementary Table1. Paired samples T-tests comparing the frequency of contact and emotional closeness between two relationship-based networks (N=32,645)

|  | | **Mean** | **SD** | ***t*** | **p-value** |
| --- | --- | --- | --- | --- | --- |
| Contact frequency ^a^ | Close-family | 1.72 | 1.12 | 125.1 | 0.000 |
|  | Other-network | 1.21 | 0.77 |  |  |
| Emotional closeness ^a^ | Close-family | 2.61 | 0.69 | 127.6 | 0.000 |
|  | Other-network | 1.47 | 0.36 |  |  |

^a^ Source: SHARE Wave 6 Social Network Module.Supplementary Table 2. Predictors of self-protective behaviors with two relationship-based network exposure measures among Europeans aged 50 and older: OLS regressions.

| **Variables** | **Self-protective behaviors (log)** | |
| --- | --- | --- |
|  | **Model 1** | **Model 2** |
| Country (ref: Germany) |  |  |
| Sweden | -0.116*** | -0.116*** |
| Spain | 0.088*** | 0.088*** |
| Italy | 0.095*** | 0.095*** |
| France | 0.009 | 0.009 |
| Denmark | -0.100*** | -0.100*** |
| Greece | 0.007 | 0.007 |
| Switzerland | -0.036*** | -0.036*** |
| Belgium | 0.010 | 0.010 |
| Israel | -0.015* | -0.015* |
| Czech Republic | 0.006 | 0.007 |
| Poland | 0.050*** | 0.050*** |
| Luxembourg | 0.038*** | 0.038*** |
| Hungary | 0.015** | 0.015** |
| Portugal | 0.032*** | 0.032*** |
| Slovenia | 0.055*** | 0.055*** |
| Estonia | -0.078*** | -0.078*** |
| Croatia | -0.023*** | -0.023*** |
| Lithuania | 0.042*** | 0.042*** |
| Bulgaria | -0.032*** | -0.032*** |
| Cyprus | -0.001 | -0.001 |
| Finland | -0.088*** | -0.088*** |
| Latvia | -0.081*** | -0.081*** |
| Malta | 0.021*** | 0.021*** |
| Romania | 0.029*** | 0.029*** |
| Slovakia | -0.060*** | -0.060*** |
| Background |  |  |
| Age - 50-59 ^a^ | -0.022*** | -0.022*** |
| Age - 70+ ^a^ | -0.013* | -0.012* |
| Gender (Female) | 0.098*** | 0.098*** |
| Education | 0.060*** | 0.060*** |
| Financial capacity | -0.032*** | -0.032*** |
| Self-rated health - baseline | -0.018** | -0.018** |
| Change in self-rated health | 0.029*** | 0.029*** |
| Marital status (Live-in partner) | 0.089*** | 0.089*** |
| Big Five personality traits |  |  |
| Openness | 0.013* | 0.013* |
| Conscientiousness | 0.038*** | 0.038*** |
| Extraversion | 0.009 | 0.009 |
| Agreeableness | 0.007 | 0.007 |
| Neuroticism | 0.035*** | 0.035*** |
| COVID-19 exposure |  |  |
| Self-exposure severity | 0.020*** | 0.019*** |
| Close-family-exposure severity | 0.014* | 0.005 |
| Other-network-exposure severity | 0.018** | 0.009 |
| Interaction terms |  |  |
| Age 50-59* Close-family-exposure severity |  | 0.013 |
| Age 70+* Close- family -exposure severity |  | 0.004 |
| Age 50-59* Other-network-exposure severity |  | 0.002 |
| Age 70+* Other-network-exposure severity |  | 0.015* |
| Observation | 33,053 | 33,053 |
| R-squared | 0.106 | 0.106 |

*Note*. COVID-19 = Coronavirus Disease 2019. *** p<0.001, ** p<0.01, * p<0.05.

^a^ Reference category: age - 60-69.Supplementary Table 3. Predictors of self-protective behaviors with cumulative country COVID-19 death rates among Europeans aged 50 and older: OLS regressions

| **Variables** | **Self-protective behaviors (log)** | |
| --- | --- | --- |
|  | **Model 1** | **Model 2** |
| Death rate in country | 0.075*** | 0.075*** |
| Background |  |  |
| Age - 50-59 ^a^ | -0.022*** | -0.023*** |
| Age - 70+ ^a^ | -0.026*** | -0.026*** |
| Gender (Female) | 0.094*** | 0.094*** |
| Education | 0.007 | 0.007 |
| Financial capacity | -0.073*** | -0.073*** |
| Self-rated health - baseline | -0.024*** | -0.024*** |
| Change in Self-rated health | 0.031*** | 0.031*** |
| Marital status (Live-in partner) | 0.105*** | 0.105*** |
| Big Five personality traits |  |  |
| Openness | 0.025*** | 0.025*** |
| Conscientiousness | 0.050*** | 0.050*** |
| Extraversion | -0.015** | -0.015** |
| Agreeableness | -0.009 | -0.009 |
| Neuroticism | 0.053*** | 0.053*** |
| COVID-19 exposure |  |  |
| Self-exposure severity | 0.017** | 0.017** |
| Close-family-exposure severity | 0.005 | -0.005 |
| Other-network-exposure severity | 0.012* | 0.001 |
| Interaction terms |  |  |
| Age 50-59* Close-family-exposure severity |  | 0.018* |
| Age 70+* Close-family-exposure severity |  | 0.001 |
| Age 50-59* Other-family-exposure severity |  | 0.006 |
| Age 70+* Other-network-exposure severity |  | 0.014 |
| Observations | 33,053 | 33,053 |
| R-squared | 0.037 | 0.037 |

*Note*. COVID-19 = Coronavirus Disease 2019. *** p<0.001, ** p<0.01, * p<0.05.

^a^ Reference category: age - 60-69.

Supplementary Table 4. Predictors of self-protective behaviors without country variable among Europeans aged 50 and older: OLS regressions

| **Variables** | **Self-protective behaviors (log)** | |
| --- | --- | --- |
|  | **Model 1** | **Model 2** |
| Background |  |  |
| Age - 50-59 ^a^ | -0.023*** | -0.024*** |
| Age - 70+ ^a^ | -0.024*** | -0.023*** |
| Gender (Female) | 0.092*** | 0.092*** |
| Education | -0.002 | -0.002 |
| Financial capacity | -0.061*** | -0.061*** |
| Self-rated health - baseline | -0.019** | -0.019** |
| Change in Self-rated health | 0.034*** | 0.033*** |
| Marital status (Live-in partner) | 0.103*** | 0.103*** |
| Big Five personality traits |  |  |
| Openness | 0.024*** | 0.024*** |
| Conscientiousness | 0.052*** | 0.051*** |
| Extraversion | -0.019*** | -0.019*** |
| Agreeableness | -0.006 | -0.006 |
| Neuroticism | 0.056*** | 0.056*** |
| COVID-19 exposure |  |  |
| Self-exposure severity | 0.019*** | 0.018** |
| Close-family-exposure severity | 0.009 | 0.000 |
| Other-network-exposure severity | 0.027*** | 0.016 |
| Interaction terms |  |  |
| Age 50-59* Close-family-exposure severity |  | 0.017* |
| Age 70+* Close-family-exposure severity |  | -0.000 |
| Age 50-59* Other-family-exposure severity |  | 0.005 |
| Age 70+* Other-network-exposure severity |  | 0.015* |
| Observations | 33,053 | 33,053 |
| R-squared | 0.032 | 0.032 |

*Note*. COVID-19 = Coronavirus Disease 2019. *** p<0.001, ** p<0.01, * p<0.05.

^a^ Reference category: age - 60-69.
